# Expression of Concern: Gut microbiota control host lipid deposition through HDAC9-driven PPARγ acetylation

**DOI:** 10.1093/ismejo/wrag164

**Published:** 2026-07-27

This is an expression of concern regarding: Yunxia Li, Qi Yuan, Yapeng Yang, Leli Wang, Qi Han, Yan Yang, Jingjing Zhang, Hong Wei, Yulong Yin, Shiyu Tao, Jie Yin, Gut microbiota control host lipid deposition through HDAC9-driven PPARγ acetylation, *The ISME Journal*, 2026, wrag109, https://doi.org/10.1093/ismejo/wrag109

Following publication of this article, the authors alerted the journal to a critical error in the one of the formulas used in Figure 5 in this paper. The journal is investigating these concerns in line with COPE guidance. In the interim, the Editors are publishing this Expression of Concern to alert readers while the outcome of the investigation is pending and advises readers to examine the details of this study with particular care.

